# Partnering bevacizumab with irinotecan as first line-therapy of metastatic colorectal cancer improves progression free survival-A retrospective analysis

**DOI:** 10.1371/journal.pone.0248922

**Published:** 2021-04-28

**Authors:** Calin Cainap, Rodica Ana Ungur, Ovidiu-Vasile Bochis, Patriciu Achimas, Catalin Vlad, Andrei Havasi, Andreea Vidrean, Anca Farcas, Tiberiu Tat, Alexandra Gherman, Andra Piciu, Madalina Bota, Anne-Marie Constantin, Laura Ancuta Pop, Dana Maniu, Ovidiu Crisan, Cosmin Vasile Cioban, Ovidiu Balacescu, Ovidiu Coza, Loredana Balacescu, Monica Mihaela Marta, Eleonora Dronca, Simona Cainap

**Affiliations:** 1 Ion Chiricuta Institute of Oncology, Cluj-Napoca, Romania; 2 Department of Oncology, Iuliu Hatieganu University of Medicine and Pharmacy, Cluj-Napoca, Romania; 3 Department of Medical Specialties, Iuliu Hatieganu University of Medicine and Pharmacy, Cluj-Napoca, Romania; 4 Department of Mother and Child, Iuliu Hatieganu University of Medicine and Pharmacy, Cluj-Napoca, Romania; 5 Department of Morphological Sciences, Iuliu Hatieganu University of Medicine and Pharmacy, Cluj-Napoca, Romania; 6 Department of Molecular Sciences, Iuliu Hatieganu University of Medicine and Pharmacy, Cluj-Napoca, Romania; 7 Faculty of Physics, Babes-Bolyai University, Cluj-Napoca, Romania; 8 Faculty of Pharmacy, Iuliu Hatieganu University of Medicine and Pharmacy, Cluj-Napoca, Romania; 9 Faculty of Dental Medicine, Iuliu Hatieganu University of Medicine and Pharmacy, Cluj-Napoca, Romania; 10 Department of Medical Education, Iuliu Hatieganu University of Medicine and Pharmacy, Cluj-Napoca, Romania; Goethe University Hospital Frankfurt, GERMANY

## Abstract

Colorectal cancer remains one of the most frequent malignancies (third place at both genders) worldwide in the last decade, owing to significant changes in modern dietary habits. Approximately half of the patients develop metastases during the course of their disease. The available therapeutic armamentarium is constantly evolving, raising questions regarding the best approach for improving survival. Bevacizumab remains one of the most widely used therapies for treating metastatic colorectal cancer and can be used after progression. This study aimed to identify the best chemotherapy partner for bevacizumab after progression. We performed a retrospective analysis of patients with metastatic colorectal cancer who were treated with bevacizumab as first- and second-line chemotherapy. Data were collected for 151 patients, 40 of whom were treated with double-dose bevacizumab after the first progression. The two standard chemotherapy regimens combined with bevacizumab were FOLFIRI/CAPIRI and FOLFOX4/CAPEOX. The initiation of first-line treatment with irinotecan-based chemotherapy improved progression-free survival and time to treatment failure but not overall survival. After the first progression, retreatment with the same regimen as that used in the induction phase was the best approach for improving overall survival (median overall survival: 46.5 vs. 27.0 months for the same vs. switched strategy, respectively). No correlations were observed between the dose intensity of irinotecan, oxaliplatin, 5-fluorouracil, or bevacizumab and the overall survival, progression-free survival in the first-/second-line treatment, and time to treatment failure. Interaction between an irinotecan-based regimen as a second-line treatment and double-dose bevacizumab after progression was associated with an improved overall survival (p = 0.06). Initiating systemic treatment with an irinotecan-based regimen in combination with bevacizumab improved the progression-free survival in the first-line treatment and time to treatment failure. In terms of overall survival, bevacizumab treatment after the first progression is better partnered with the same regimen as that used in the induction phase.

## Introduction

Colorectal cancer (CRC) represents one of the most challenging malignancies of the digestive tract. Due to inherent changes in our diet, nutritional habits, and the way in which food is preserved and prepared, the general trend is an increase in the incidence of this type of cancer. The incidence of CRC has been reported to be 19.7 cases per 100,000 persons worldwide, similar to the incidence of lung cancer (22.5 cases per 100,000 persons) [1, 2]. In 2018, almost half a million new cases of CRC were diagnosed in Europe. In Romania, the incidence of CRC was 13.3% (> 11,000 new cases), which is the second highest incidence after that of lung cancer (13.6%; 11,300 new cases in 2018) [1, 2], followed by gastric, liver, and pancreatic cancer [1–3].

The prognosis of CRC varies greatly depending on the disease stage at diagnosis. Similar to breast and prostate cancer, CRC also has an increasing number of long-term survivors [4]. Approximately 95% of the deaths caused by CRC occur within the first5 years after the initial diagnosis [4]. Between 30% and 50% of patients with CRC develop a recurrence (loco regional or distant) [5].

Even in the metastatic stage, the prognosis of CRC is promising and may continue to improve as new drugs and treatment strategies are developed. Currently, patients with metastatic CRC (mCRC) can benefit from standard chemotherapy as well as anti-epidermal growth factor receptor and anti-angiogenic targeted therapies. These therapeutic advancements have fundamentally changed the natural history of mCRC. The most effective treatment strategies can now yield a mean survival time of 30 months compared with the previous 3–6 months. Given the wide range of drugs available, oncologists face difficult medical decisions that must take into consideration not only the efficacy of a drug but also the patients’ quality of life, treatment compliance, drug toxicity, comorbidities, and potential allergic reactions [6, 7]. The clinical strategy for mCRC resembles that of solving a complicated puzzle and includes indications for surgery, oligometastatic or plurimetastatic diseases, tumor responses to treatment (especially after treatment with curative intent), and disease-free intervals. Thus, despite the low cure rate, mCRC must be regarded as a chronic disease requiring all available therapeutic options to be used individually or in combination for improved patient benefit and better survival outcomes.

Bevacizumab was approved for mCRC due to its survival advantage when combined with chemotherapy. Its efficacy may be influenced by the accompanying chemotherapeutic agent. Therefore, we aimed to conduct a study of patients with mCRC who were treated with bevacizumab in first- and second-line settings (after progression) to examine the impact of the chemotherapy partner on overall survival (OS), progression-free survival in the first-line treatment (PFS1), progression-free survival in the second-line treatment (PFS2), and time to treatment failure (TTF). Our data showed that first-line irinotecan-based chemotherapy combined with bevacizumab improved PFS1 and TTF. After the first disease progression, doubling the dose of bevacizumab and maintaining the same chemotherapy regimen as that used in the induction phase improved OS. No significance was identified for PFS2 regarding the chemotherapy partner for bevacizumab.

## Materials and methods

### Study design

The Institute of Oncology Cluj-Napoca is one of the largest medical institutions in Romania, where patients from all over the country are treated. We searched the medical records of our institution to identify patients who were treated for CRC between January 2009 and December 2017. The initial group included 5649 patients who received at least two lines of chemotherapy, as per their medical records. We then selected those patients (n = 694) who received bevacizumab as part of their treatment. Of these, only 162 patients were treated with bevacizumab after the first progression. Eleven patients received more than two lines of chemotherapy (according to European recommendations) and were excluded from the analysis. Of the remaining 151 patients, 40 were treated with a double dose of bevacizumab (DDB) after progression and 111 were treated with a standard dose of bevacizumab (SDB).

### Informed consent

The study design was approved by the Ethics Committee of the Institute of Oncology Cluj-Napoca, Romania (approval number: 42/8 December 2015). Research was conducted in accordance with the Declaration of Helsinki. All patients provided written informed consent before commencing treatment. All data were anonymized before the commencement of the study, as per the General Data Protection Regulations.

### Inclusion and exclusion criteria

We adopted the standard inclusion criteria that are used for this type of pathology in most clinical trials [7–10]. Moreover, we used retrospectively collected data from consecutively treated patients.

The inclusion criteria were: age 18 or above, histological confirmed CRC, lab tests adequate for chemotherapy: neutrophil count > 1.5 x 10^9^/L, leukocyte count > 3x 10^9^/L, platelet count > 100 x 10^9^/L, hemoglobin level > 9 g/dL, transaminases (AST and ALT) below 2.5X the upper limit of the normal range (ULN) in patients without liver metastases or below 5 X ULN in case of liver metastases, total bilirubin below 3 mg/dL, creatinine clearance > 50 mL/min, negative urinary protein on dipstick testing or < 1 g/24-hour collection; no medical contraindication to chemotherapy (according to treatment characteristics and recommendations), at least one metastatic site, Eastern Cooperative Oncology Group (ECOG) performance status of 0 to 2, bevacizumab administration in first- and second-line treatment, adequate follow-up (at least monthly clinical checkup and CT scan every 3–4 months).

***The exclusion criteria*** were: previous administration of chemotherapy for the metastatic stage, uncontrolled comorbidities, poor performance status (ECOG ≥ 3), inadequate lab tests, hypersensitivity to the active substance, heart failure (NYHA grade > 2), uncontrolled hypertension, acute myocardial infarction (≤ 6 months) and pregnancy.

### Patients’ characteristics

After a rigorous search of the Institute database we identified 162 patients who met general inclusion criteria. After a second check-up were identified to be treated outside general recommendation, which means administration of bevacizumab through multiple lines or beyond the third line of chemotherapy, so these patients were excluded from final population. The main characteristics of included patients are presented in Table 1.

**Table 1:** Baseline patient characteristics.

Characteristic	All (151)
**Age (years)**	
Median (range)	57 (19–75)
**Gender**	
Male	86 (57.0%)
Female	65 (43.0%)
**First-line chemotherapy**	
Oxaliplatin-based	90 (59.6%)
Irinotecan-based	60 (39.7%)
**Second-line chemotherapy**	
Oxaliplatin-based	53 (35.1%)
Irinotecan-based	97 (64.2%)
**Primary tumor site**	
Left	35 (23.2%)
Right	116 (76.8%)
**Metastasis site**	
Liver	121 (59.0%)
Lung	25 (12.3%)
Peritoneum	31 (15.1%)
Lymph nodes	8 (3.9%)
Bone	7 (3.4%)
Other	13 (6.3%)
**Number of organs with metastasis**	
1	110 (72.8%)
> 1	41 (27.2%)

Data are number (%) unless otherwise specified.

### Treatments

The chemotherapy regimens used at our institution included FOLFIRI (5-fluorouracil, leucovorin, and irinotecan), FOLFOX4 (5-fluorouracil, leucovorin, and oxaliplatin), CAPIRI (capecitabine in combination with irinotecan), and CAPEOX (capecitabine in combination with oxaliplatin) in standard doses. All the dose modifications conformed to our institution’s protocols [7].

**Fig 1:**
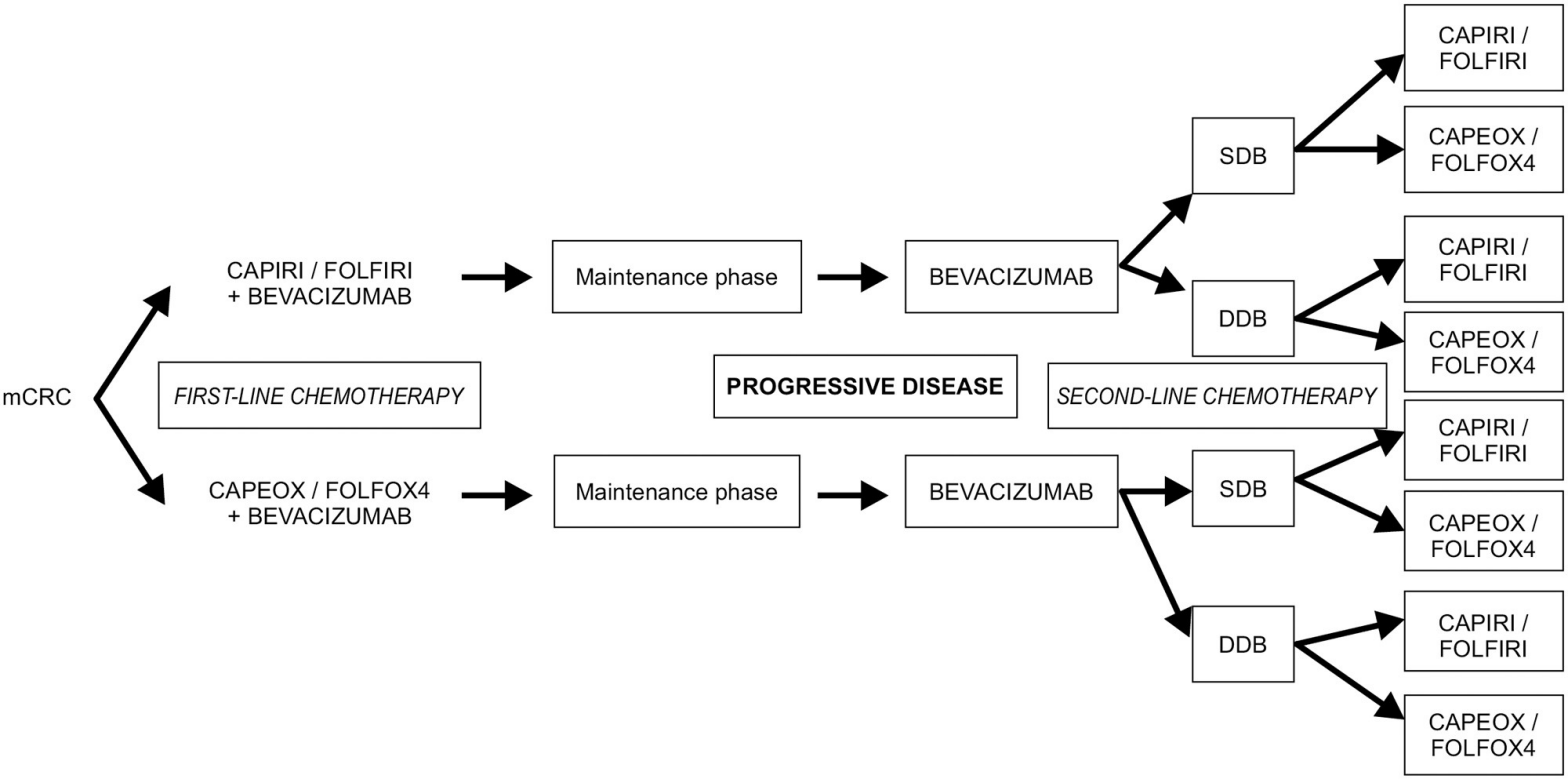
Treatment strategies for the metastatic colorectal cancer patients included in this study. DDB, double-dose bevacizumab; mCRC, metastatic colorectal cancer; SDB, standard-dose Bevacizumab.

In the first-line treatment, the bevacizumab dose was standardized and there was no difference between the SDB and DDB groups (the intensity of the dose was the same: 2.5 mg/kg per week). A dose of 5.0 mg/kg bevacizumab was administered if at 2 weeks (in combination with FOLFIRI or FOLFOX4) and 7.5 mg/kg if at 3 weeks (in combination with CAPEOX or CAPIRI). Dose adjustments were permitted provided they were performed in accordance with the institutional protocols. At the first progression, the dose of bevacizumab was doubled in the DDB group. The therapeutic strategy for patients with mCRC from a systemic treatment perspective is presented in Fig 1. Only patients who were treated according to this workflow were included in this study. All included patients had an Eastern Cooperative Oncology Group performance status of 0–2, and no significant differences were noted between groups.

Before each chemotherapy cycle, patients underwent a medical examination, blood sampling for hematology and biochemistry, and urinalysis for proteinuria. Imaging evaluation was performed every 3–6 months to assess the tumor response to systemic treatment.

### Statistical analysis

Descriptive statistics were evaluated using two tests. The Kaplan–Meier method was used to construct OS, PFS1, PFS2, and TTF curves. OS was defined as the time interval between the first cycle of chemotherapy and the date of death. TTF was defined as the time interval between the first cycle of chemotherapy and the last cycle of second-line chemotherapy with bevacizumab (i.e., time to second progression). PFS1 was defined as the time interval between the first cycle of chemotherapy and the last cycle of first-line chemotherapy. PFS2 was defined as the time interval between the first cycle of chemotherapy and the last cycle of second-line chemotherapy.

A log-rank test was used to determine differences between the survival curves. Hazard ratios (HRs) and 95% confidence intervals (CIs) were estimated using a Cox regression. We calculated Pearson correlation coefficients to explore the relationships between the main outcome measures. Statistical analyses were conducted using Excel 2010 and R version 3.5.1 Microsoft Windows version 7. Statistical significance was set at a two-tailed p-value of < 0.05.

## Results

In a previous study, we presented the results of a retrospective analysis of patients with mCRC who were treated with bevacizumab after progression [7]. DDB at first progression was shown to have improved OS (41 vs. 25 months, log-rank p = 0.01; HR: 0.62 [95% CI: 0.42–0.91], p = 0.02) and TTF (24 vs. 19 months, log-rank p<0.01; HR: 0.61 [95% CI: 0.42–0.89], p = 0.09) compared with SDB after progression [7].

In the first-line treatment, the dose of bevacizumab was standardized and there were no differences between the SDB and DDB groups (the intensity of the dose was the same: 2.5 mg/kg per week). The main difference was in the backbone chemotherapy regimen (oxaliplatin- or irinotecan-based regimens in the first-line treatment).

The outcomes of the study (OS, PFS1, and TTF) were compared between standard chemotherapy regimens as a first-line treatment, given that bevacizumab was administered to all patients and the dose intensity of the first-line treatment was the same. The PFS1 and TTF were significantly better in patients who received irinotecan-based chemotherapy as a first-line treatment (CAPIRI/FOLFIRI: HR: 0.69 [95% CI: 0.49–0.95], p = 0.03) than in those who did not (Table 2).

**Table 2:** Progression-free survival in the first-line setting according to the backbone chemotherapy regimen.

PFS1 (months)	Median	Lower 95% CI	Upper 95% CI	p-value	HR (95% CI)
**CAPIRI/FOLFIRI + bevacizumab**	15	12	18	0.02	0.69 (0.49–0.95)
					p = 0.03
**CAPEOX/FOLFOX4 + bevacizumab**	12	10	13		

Abbreviations: CI–confidence interval, HR, hazard ratio; PFS1, progression-free survival in the first-line setting.

**Fig 2:**
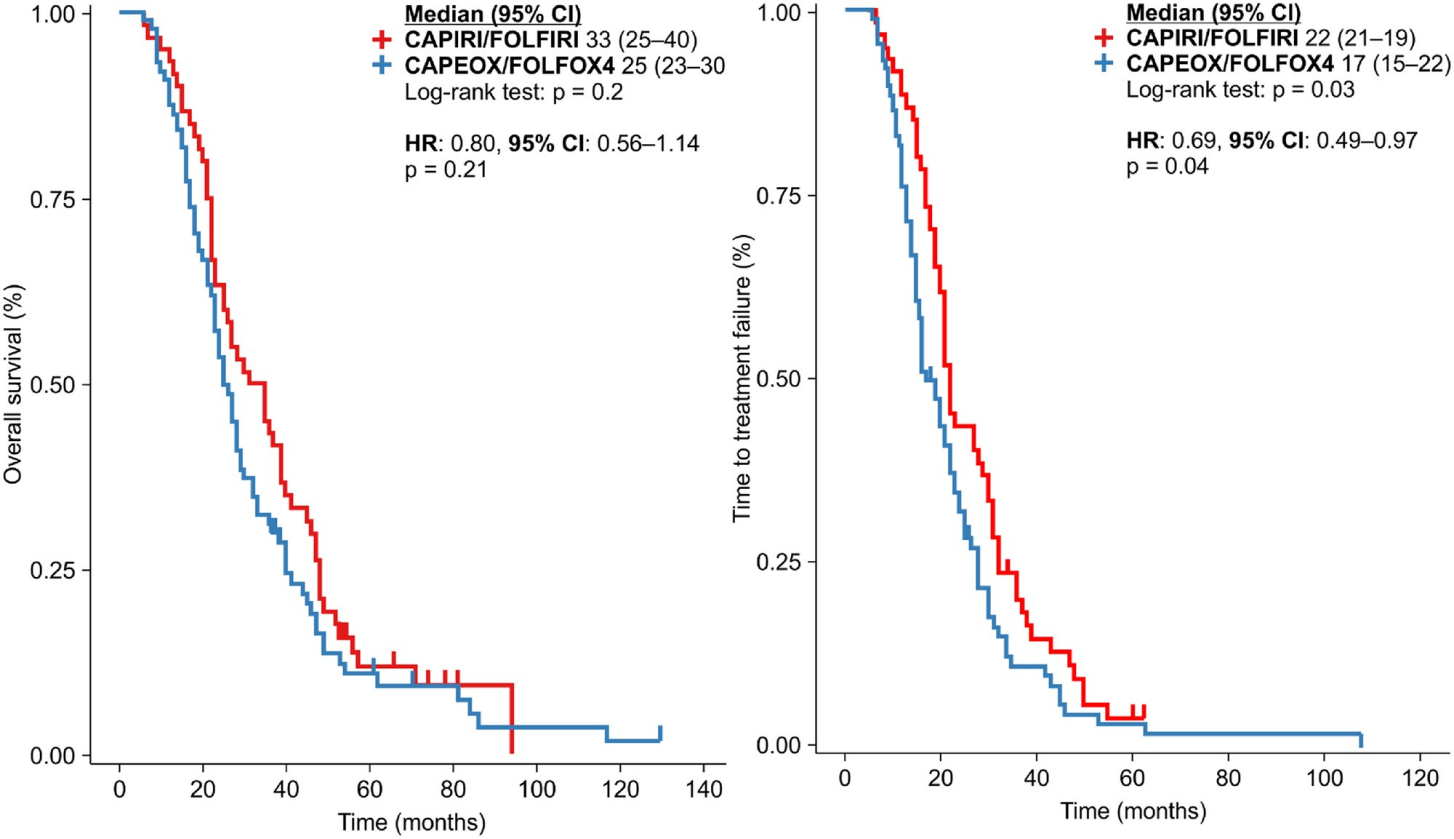
Overall survival and time to treatment failure for all patients in line with the backbone chemotherapy used: CAPIRI/FOLFIRI vs. CAPEOX/FOLFOX4.

No significant differences in OS were observed between the oxaliplatin- and irinotecan-based regimens in the first-line treatment, irrespective of the dose of bevacizumab (Fig 2).

**Fig 3:**
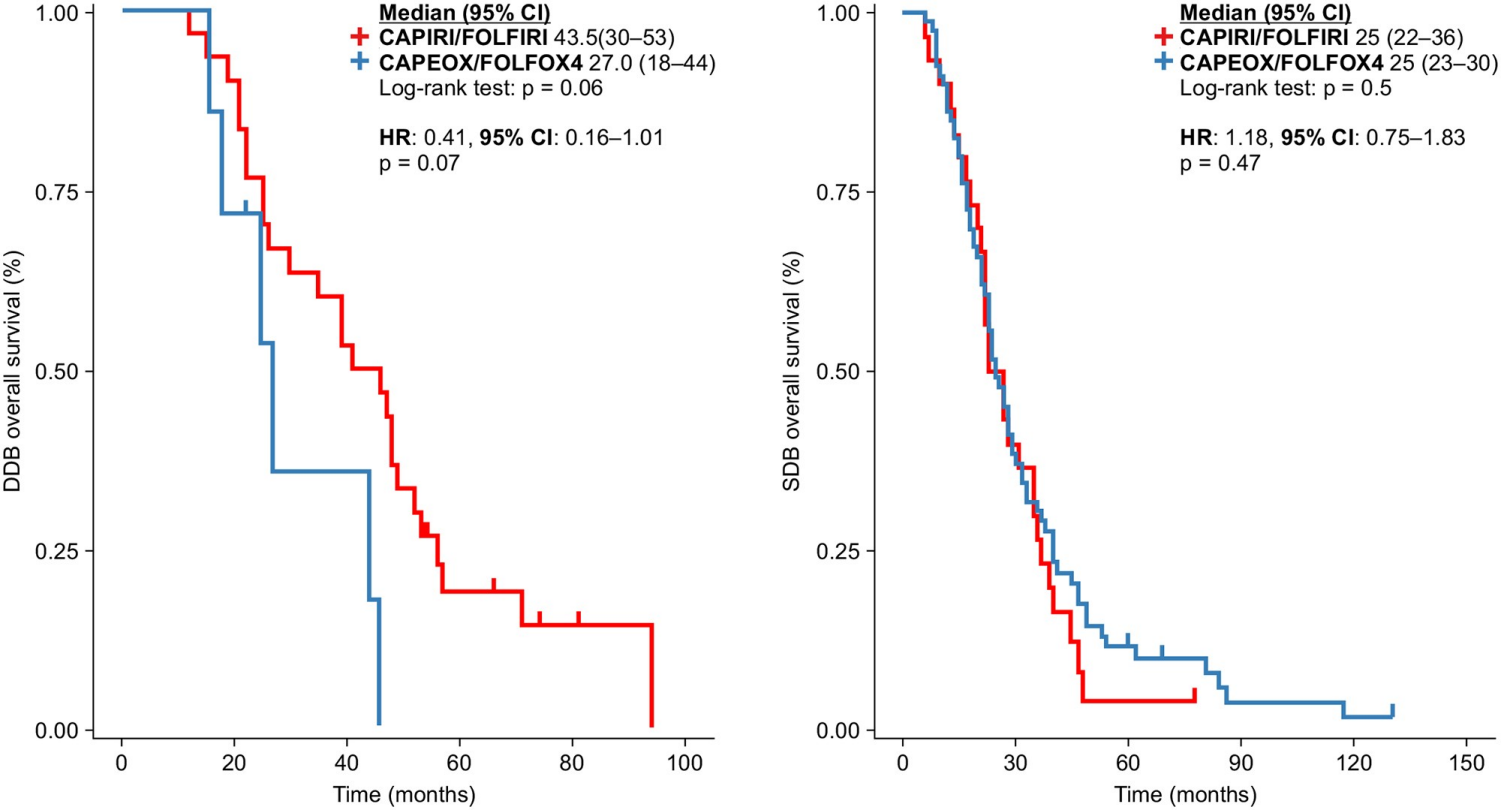
Overall survival, according to the first-line backbone chemotherapy in the double-dose and standard-dose bevacizumab groups.

The TTF calculated for all patients included in this study revealed a survival advantage for patients treated with an irinotecan-based regimen in the first-line setting compared to those treated with an oxaliplatin-based regimen (TTF: 22 vs. 17 months, p = 0.03; HR: 0.69 [95% CI: 0.49–-0.97], p = 0.04) (Fig 3).

Because there were differences in the dose intensity of bevacizumab after the first progression in the second-line treatment, an analysis was performed to assess TTF between the SDB and DDB groups. Patients in the DDB group had a significant advantage in TTF (TTF: 24 vs. 19 months, p<0.01; HR: 0.61, p<0.01) (Table 3).

**Table 3:** Time to treatment failure in the double-dose and standard-dose bevacizumab groups.

TTF (months)	Total	Failed	Censored	Median	Lower 95% CI	Upper 95% CI
**DDB group**	40	37	3	24	21	35
**SDB group**	111	103	8	19	16	22

**Fig 4:**
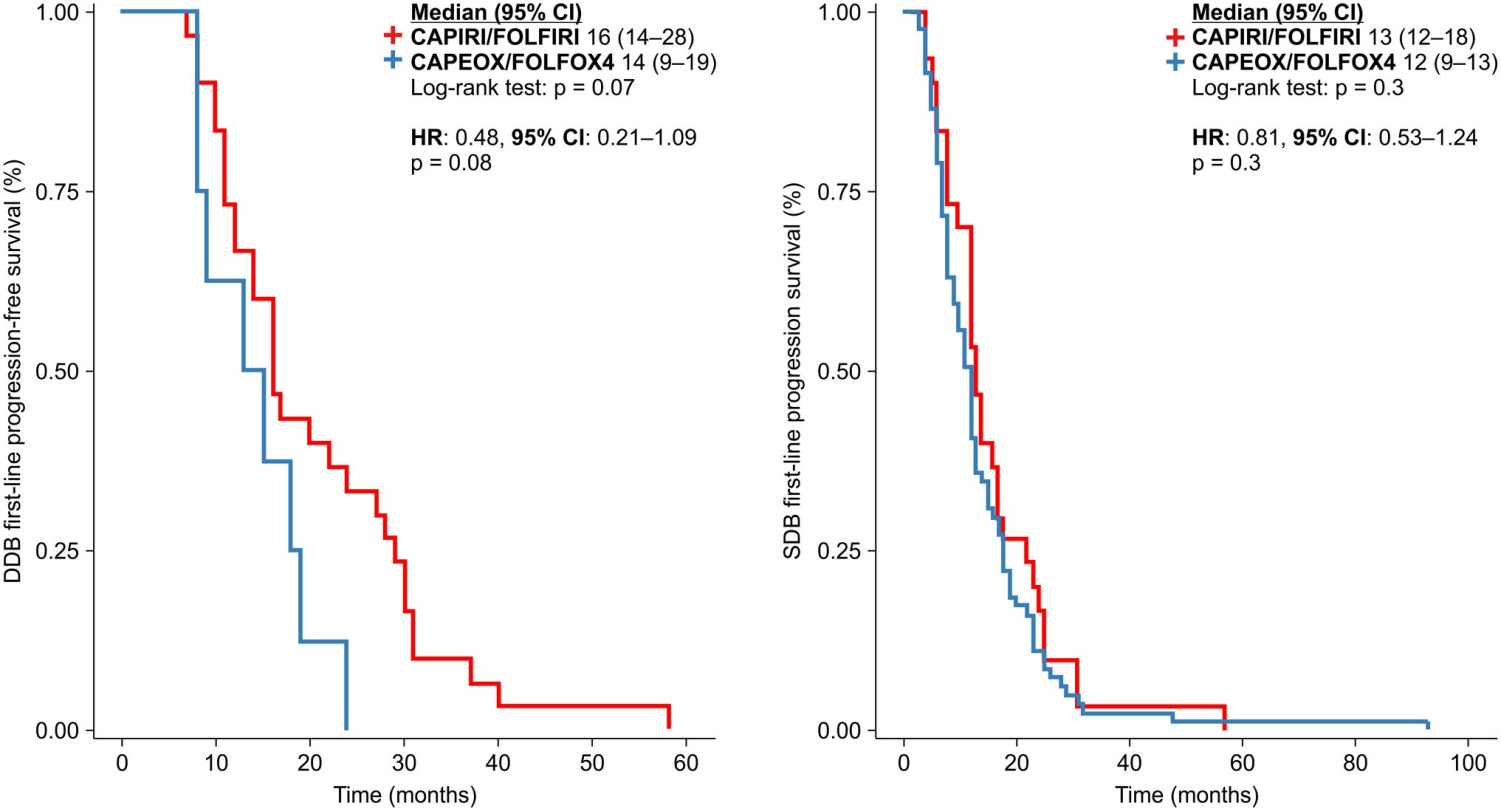
Progression-free survival in the first-line treatment of patients in the double-dose and standard-dose bevacizumab groups.

In a previous study, we showed that patients receiving DDB after progression had a better OS [7]. In this study, we also analyzed whether the choice of first-line treatment influenced the OS in the SDB and DDB groups. No significant differences were observed between the two groups. However, there was a trend toward a better OS in patients receiving irinotecan-based regimens in the DDB group than those in the SDB group (43.5 vs. 27.0 months, p = 0.06; HR: 0.41 [95% CI: 0.16–1.01], p = 0.07) (Fig 4).

Analysis of all patients revealed a better PFS1 in those receiving irinotecan-based chemotherapy than in those receiving oxaliplatin-based chemotherapy. Bevacizumab administered in combination with standard chemotherapy as a first-line treatment was the same, irrespective of the patient group (SDB or DDB). When the analysis was split, no significant differences were observed in either group. In the DDB group, the HR was 0.48 (p = 0.08). The median PFS1 for CAPIRI/FOLFIRI vs. oxaliplatin was 16 vs. 14 months, respectively in the DDB group (p = 0.07) and 13 vs.12 months, respectively in the SDB group (p = 0.3); the HR was 0.81 [95% CI: 0.53–1.24] (p = 0.3) (Fig 4).

**Fig 5:**
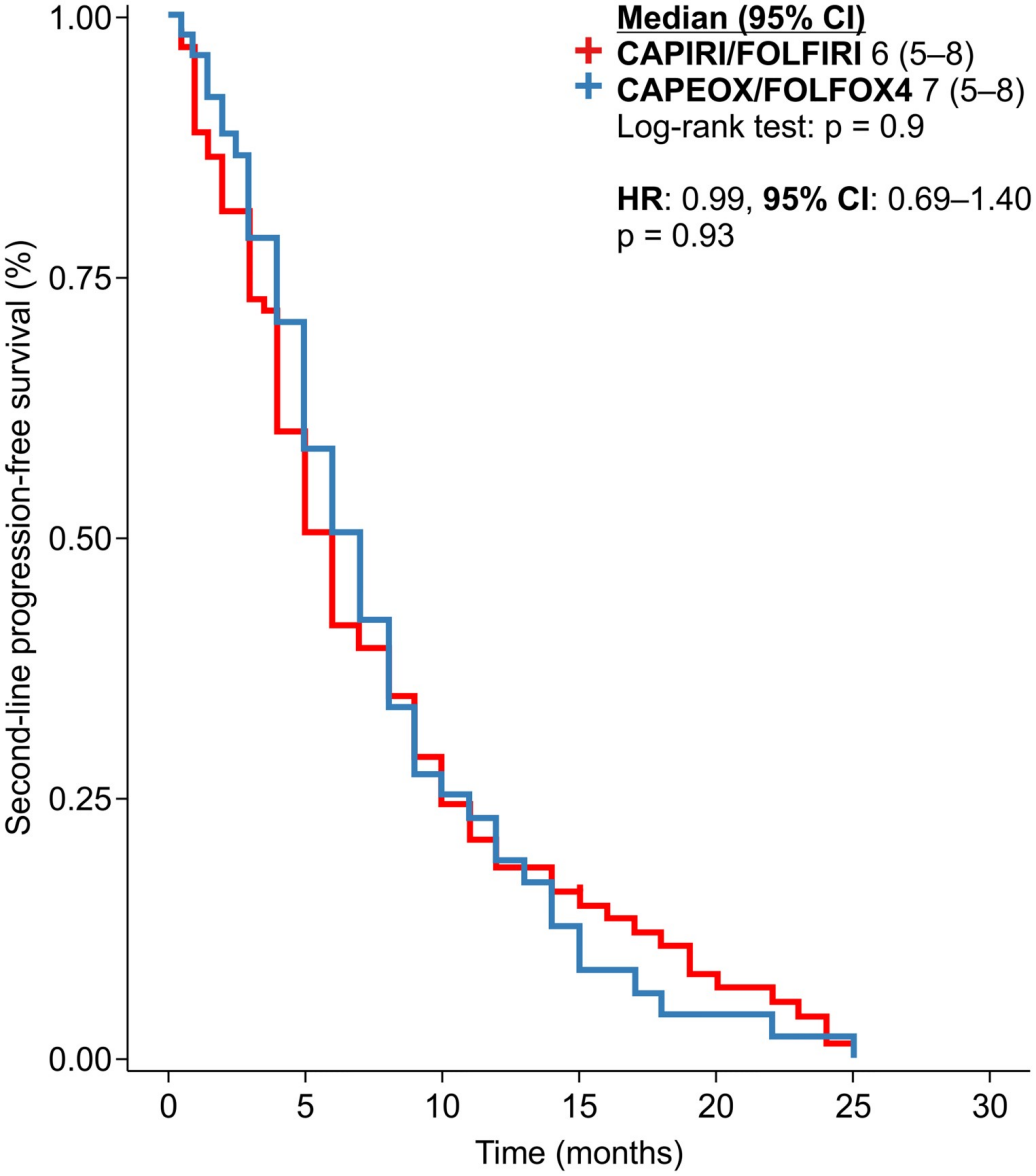
Progression-free survival in the second-line treatment setting in line with the backbone chemotherapy, irrespective of bevacizumab dose.

In the second-line treatment, we investigated how PFS2 could be modified by the standard chemotherapy regimen used, irrespective of the dose of bevacizumab. No significant differences were observed (Fig 5).

**Fig 6:**
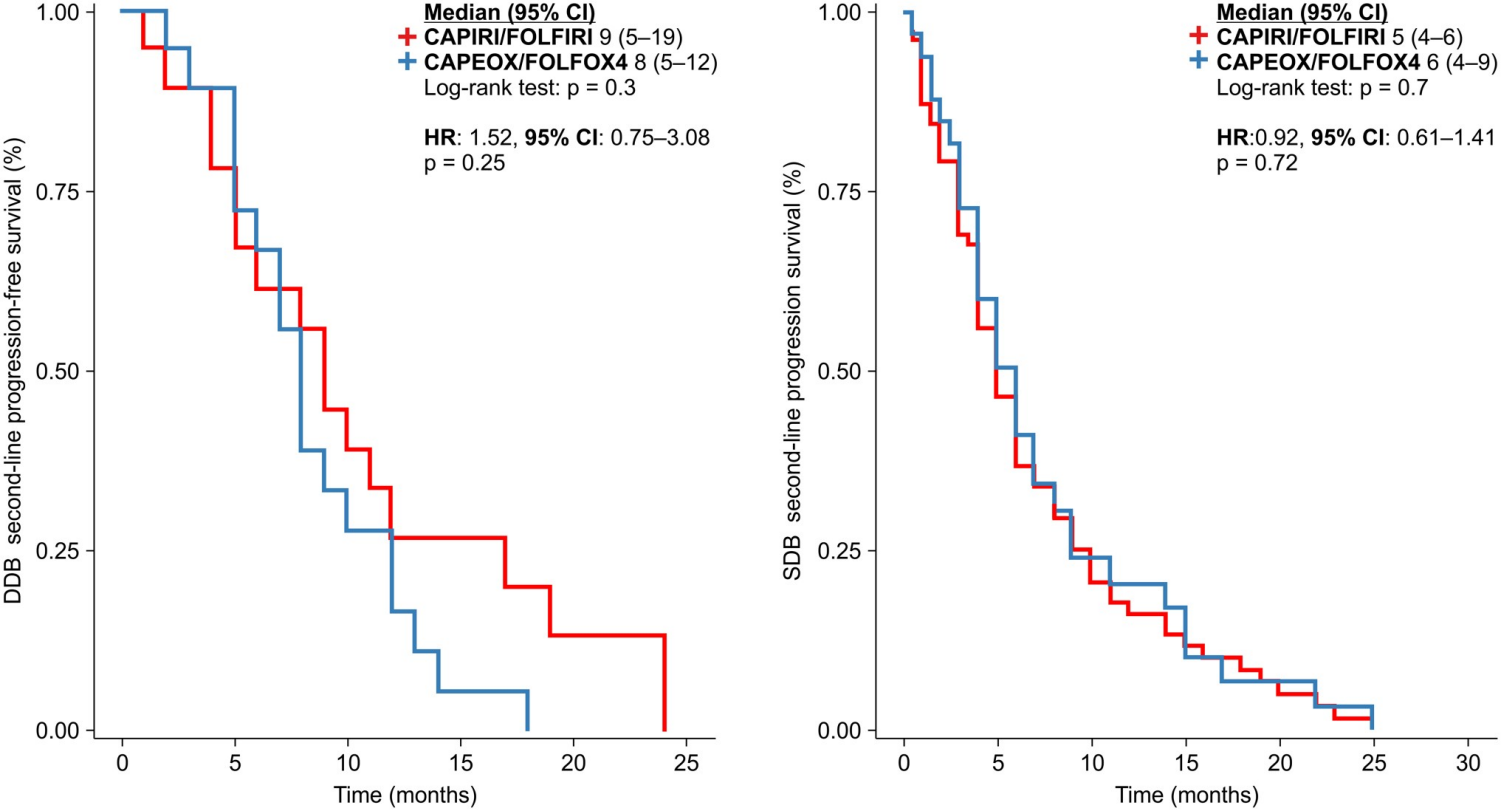
Progression-free survival in the second-line treatment of patients in the double-dose and standard-dose bevacizumab groups.

Because the dose of bevacizumab administered after the first progression differed between the groups, we analyzed whether the backbone chemotherapy regimen could modify the PFS2. However, no significant differences were observed (Fig 6).

Given that different doses of bevacizumab were administered in the first-line treatment and after the first progression, we calculated the Pearson correlation coefficients between the OS, PFS1, PFS2, and TTF and the dose intensities of the CAPIRI/FOLFIRI-, CAPEOX/FOLFOX4-, and bevacizumab-based regimens in the first-and second-line settings for the entire cohort and for patients in the SDB and DDB groups. No significant correlations were observed.

Considering the inconsistencies in recommendations from published clinical trials on whether or not to change the chemotherapy partner for bevacizumab at first progression, we investigated the clinical practice and outcomes at our institute, as detailed in Fig 1.

Of the 151 patients included in this study, we had data for 149 patients. The treatment changes are summarized in Table 4. In 14 (approximately 10%) of the 149 patients, the standard chemotherapy partner was not changed at first progression.

**Table 4:** Summary of treatment changes at first progression.

First-vs. second-line treatment	Total	DDB	SDB
**Irinotecan-based followed by oxaliplatin-based therapy**	48	18	30
**Oxaliplatin-based followed by irinotecan-based chemotherapy**	87	7	80
**Irinotecan-based chemotherapy in both lines of treatment**	12	12	0
**Oxaliplatin-based chemotherapy regimen in both lines of treatment**	2	1	1
**Total**	149	38	111

**Fig 7:**
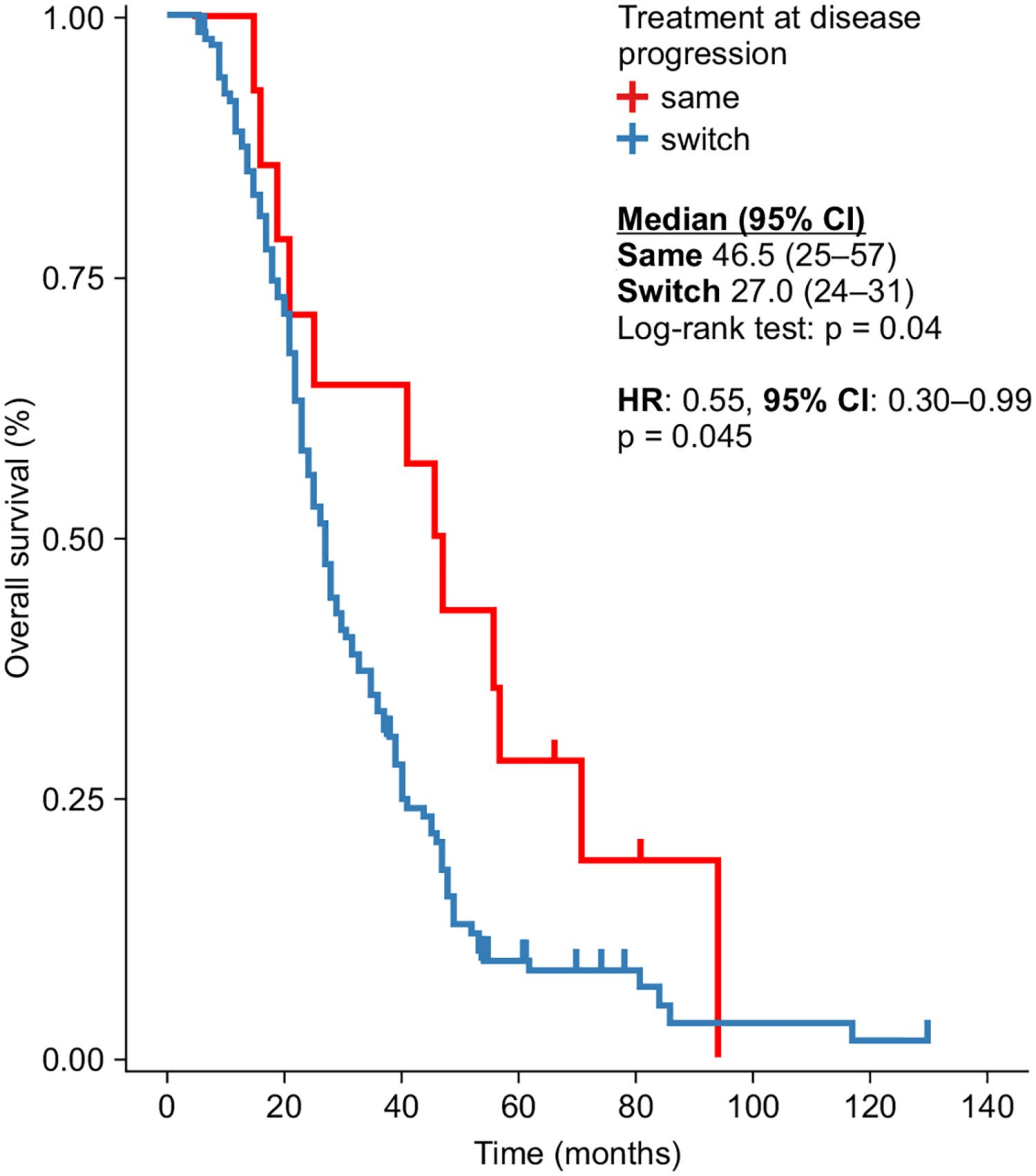
Overall survival in line with the concordance of first- and second-line treatment with bevacizumab dose.

Retaining the chemotherapy regimen associated with bevacizumab after the first progression had a significant OS advantage (46.5 vs. 27 months, log-rank p = 0.04). The HR (0.55) favored the same regimen at the first progression (p = 0.04) (Fig 7).

In our analysis, patients treated with an irinotecan-based regimen as the first-line treatment and who were treated with the same regimen after the first progression had a better median TTF than did those who were treated with an oxaliplatin-based regimen after the first progression (Table 5). However, no significant differences were observed between the SDB and DDB groups (HR: 1.016 [95% CI: 0.51–2.02], p = 0.96).

**Table 5:** Time to treatment failure according to the chemotherapy partner after first progression (same vs. switched regimens).

Strategy at first progression	First-line CT	Second-line CT	TTF (months), median (95% CI)	p-value (log-rank)	HR (95% CI)	p-value (log-rank)
**Same regimen**	FOLFIRI/CAPIRI	FOLFIRI/CAPIRI	29 (20–43)	0.04	0.18 (0.03–1.06)	0.058
	FOLFOX4/CAPEOX	FOLFOX4/CAPEOX	17 (15–19)			
**Switched regimen**	FOLFIRI/CAPIRI	FOLFOX4/CAPEOX	21 (20–29)	0.1	0.75 (0.52–1.08)	0.12
	FOLFOX4/CAPEOX	FOLFIRI/CAPIRI	17 (15–23)			

No significant differences in the TTF were observed in relation to the switched strategies.

We investigated potential differences in the TTF and PFS2 using Pearson correlation coefficients between the backbone chemotherapy regimen used as the first-line treatment, the dose of bevacizumab after the first progression, and the same or switched chemotherapy regimen after the first progression. However, none of these correlations were significant.

In our study, irinotecan-based chemotherapy administration as the first-line treatment for mCRC patients treated with bevacizumab beyond progression improved PFS1 and TTF, but not OS or PFS2. Re-intensification of treatment with the same regimen as was utilized in the induction phase improved OS.

## Discussion

Only a small proportion of patients with mCRC will achieve an adequate tumor response to curative treatment. In the majority of patients, the aim is to maintain tumor control through systemic treatment with palliative intent, where a continuum of care is the main goal of therapy. One of the most commonly used systemic regimens is oxaliplatin-based chemotherapy as a first-line treatment for mCRC [11].

“Which is the best choice for first-line treatment?” remains one of the most important unanswered questions regarding the optimal treatment of patients with mCRC. Before the targeted therapy era, systemic chemotherapy was the mainstay of first-line treatment. In our analysis, both the PFS1 (p = 0.02) and TTF (p = 0.03) were better if the first-line treatment administered was irinotecan-based chemotherapy, irrespective of the use of bevacizumab (SDB or DDB, after disease progression). No differences were observed in OS or PFS2 when irinotecan-based regimens were compared with oxaliplatin-based regimens as the first-line treatment. However, first-line irinotecan-based chemotherapy seemed to be a better partner for bevacizumab and improved OS (p = 0.06), especially when bevacizumab was administered as a double-dose after progression. The same trend was observed in PFS1for irinotecan-based regimens in the DDB group (p = 0.07). Doubling the dose of bevacizumab appeared to improve PFS2 more than switching the standard chemotherapy partner in the second-line treatment did [7].

There is an ongoing debate regarding the optimal use of 5-fluorouracil in standard chemotherapy regimens. The best approach is probably represented by the use of all available drugs, including irinotecan and oxaliplatin, based on the patients’ disease histories. The sequence of treatment adopted depends on the aim of the treatment because the efficacies and toxicities differ; neurotoxicity and allergic reactions are associated with oxaliplatin, whereas diarrhea and neutropenia are more common in irinotecan-based chemotherapy.

A number of randomized trials have compared irinotecan- and oxaliplatin-based regimens, with conflicting results for the PFS, response rates, and OS. However, taken together, most of them agree that oxaliplatin-based chemotherapy in the first-line treatment is associated with significantly higher response rates [6, 12, 13]. This could be beneficial if the aim of the first-line treatment is conversion to surgery.

Published meta-analyses have reported conflicting results. A meta-analysis of nine randomized studies and 3710 patients by Jang et al. [14] showed improvement in the OS and PFS in the irinotecan-treated group alone. A meta-analysis of 22 studies involving 2675 patients by Chan et al. [15] suggested a greater benefit for irinotecan-based chemotherapy than oxaliplatin-based treatment when combined with an anti-angiogenic agent such as bevacizumab or aflibercept. The Macedo et al. [16] study of six clinical trials and 3060 patients reported an OS advantage in the irinotecan-treated group when bevacizumab was added as the first-line treatment. The study of eight randomized controlled trials involving 3424 patients by Xuet al. [17] reported a better PFS and safety profile for the irinotecan, fluorouracil, and leucovorin regimen in combination with bevacizumab as well as advantages in OS and response rates for FOLFOX4. In a meta-analysis of 3763 patients done by Hurwitz et al. [18], 1990 of them were treated with bevacizumab and standard backbone chemotherapy (irinotecan- or oxaliplatin-based regimens). For patients treated as first line, only irinotecan-based regimens with bevacizumab were significantly associated with OS and PFS. Besides meta-analyses, there are also the results of published trials, such as the Avastin(^®^) Registry—Investigation of Effectiveness and Safety study on 1211 patients, in which no significant differences were observed between FOLFOX and FOLFIRI in combination with bevacizumab as a first-line treatment [19]. In the Bevacizumab Expanded Access Trial study of 1914 patients, the comparison was extended beyond the standard FOLFOX vs. FOLFIRI regimens to include fluoropyrimidine-based chemotherapy as capecitabine associated with oxaliplatin [8]. All three regimens were associated with a similar OS and PFS. In another phase III trial (CALGB/SWOG80405) of 1100 patients [9], an unplanned subgroup analysis revealed that FOLFOX may be a better partner for cetuximab, but not for bevacizumab; however, FOLFIRI was not statistically associated with either type of therapy, with the reserve of a very unbalanced arm in favor of FOLFOX (70.8% vs. 26.3% for FOLFIRI) [9]. In a phase III trial of 395 patients, Yamazaki et al. [10] investigated the efficacies of FOLFOX and FOLFIRI administered in combination with bevacizumab. The two proposed regimens were equivalent in terms of the PFS (the main outcome of the trial), OS, and response rates.

Botrel et al. [20] investigated the type of fluoropyrimidine used in combination with irinotecan or oxaliplatin when administered with bevacizumab in 3914 patients from nine randomized clinical trials. A positive effect on the PFS and OS was shown for irinotecan-based chemotherapy when administered with bevacizumab. Conversely, oxaliplatin-based chemotherapy showed no significant differences for PFS or OS. In elderly patients, the combination of bevacizumab with fluoropyrimidine was found to be more effective for OS and PFS than the addition of a third agent, such as irinotecan or oxaliplatin [21].

In this study, we investigated whether the dose intensity of the agents used for the treatment of patients with mCRC correlated with the OS, PFS1, PFS2, and TTF. The Pearson correlation coefficients for both the SDB and DDB groups after the first progression showed no significant associations between dose intensity and the outcomes investigated. The only exceptions were the OS and the dose intensity of the irinotecan-based regimen as second-line treatment with DDB after the first progression, and TTF and the dose intensity of oxaliplatin-based regimens as a first-line treatment.

In addition to efficacy, systemic treatment needs to have an acceptable toxicity profile to encourage patient adherence to the modern concept of a continuum of care. This strategy assumes that efficient drugs with low toxicity are capable of maintaining disease control and can be used as maintenance therapy and post-progression therapy.

For maintenance therapy, the clinician will often offer the patient a de-escalating treatment where one or more of the agents in the systemic treatment are withdrawn. This reduction in treatment intensity is usually maintained until disease progression, at which point the initial treatment can be intensified or the treatment regimen can be changed. In published maintenance trials, both situations have been encountered. In fact, retreatment with the same initial regimen at disease progression could be defined as administration after progression, especially when the time interval from the cessation of chemotherapy approaches the point of disease progression. Consequently, this maintenance phase reduces the dose intensity of the systemic drug used.

For oxaliplatin, neurotoxicity could be dose limiting with an obvious impairment of efficacy [6]. Similar to other chemotherapy agents, 5-fluorouracil can induce cardiotoxicity [22]. Robinson et al. [23] conducted a meta-analysis showing that irinotecan is responsible for steatohepatitis, which is a risk factor for perioperative morbidity and mortality (1 in every 12 patients treated with this regimen). Instead of non-alcoholic fatty liver disease caused by chemotherapy, oxaliplatin-based regimens seem to be responsible for sinusoidal obstruction syndrome in > 30% of patients [23]. Bevacizumab has been shown to influence the toxicity of oxaliplatin. A study by Rubbia-Brandt et al. [24] was one of the first to demonstrate that bevacizumab can reduce the incidence of severe sinusoidal obstruction syndrome. In patients who received bevacizumab as part of systemic therapy, the incidence of sinusoidal obstruction syndrome was 31.4% compared with 62.2% in those who did not receive bevacizumab [24].

Data on the cut-off intervals for safely intensifying the induction chemotherapy in patients with mCRC are scarce in the literature. For oxaliplatin-based regimens, a 6-month interval was proposed in the OPTIMOX2 trial [25, 26], and in the COntinuous or INntermittent (COIN) trial, 4 weeks was established as the platinum-refractory definition [26]. For irinotecan-based chemotherapy, a free interval of 3 months was associated with the restoration of irinotecan sensitivity [27].

In the OPTIMOX1 trial, the dose of oxaliplatin in the FOLFOX7 regimen was increased by 37% compared to that in the FOLFOX4 regimen [25, 28]. The increased dose was designed to improve outcomes. However, a disappointing rate of reintroduction of oxaliplatin per protocol (<25% had optimal oxaliplatin reintroduction; the remaining 75% experienced delays) meant that the response rates were similar between the two arms [25]. In a pooled analysis of two phase II trials, Nakayama et al. [27] demonstrated that the dose intensity of irinotecan and oxaliplatin and any delay in administration could impair the PFS. A Canadian study of 1989 patients with mCRC showed that intervals without irinotecan-based chemotherapy with bevacizumab were detrimental to OS [29].

Approximately 10% of the patients in our study who were treated with bevacizumab after progression did not have an additional change in the standard chemotherapy partner at the time of progression. In our analysis, keeping the same partner at the first progression proved to be more advantageous for OS than the switch strategy (46.5 vs. 27.0 months, p = 0.04). Initiating the first-line treatment with an irinotecan-based regimen improved the TTF compared with an oxaliplatin-based regimen. The same positive influence of irinotecan-based chemotherapy was seen when retaining the same treatment regimen in the case of progression.

For most patients, there will be a progressive phase at some point in their disease history. At that point, the oncologist and the patient have two options available to them: intensify treatment with the same regimen as in the induction phase or change the treatment.

Data are also available from maintenance trials. In the AIO 0207 trial, 472 patients were randomized for maintenance therapy, and those with active treatment had a better PFS, but not OS, compared with the observation arm [30]. The lowest probability of reinduction with the same initial regimen was observed for the bevacizumab and fluoropyrimidine maintenance group (19%), which was less than half that for the observation arm (46%) [30]. In the CAIRO 3 trial, 558 patients were randomized between the maintenance and observation arms, and the PFS2was defined as the time to second progression, which included PFS1 (the time from randomization to first progression). If PFS1 was subtracted, then, after reinduction with the same regimen, disease control was maintained for only 3.2months [31]. In the OPTIMOX2 trial, reinduction with oxaliplatin-based chemotherapy prolonged disease control for 4.8 months in the maintenance group compared with 3.9 months for the complete-free interval [25]. The time to disease control (induction plus reinduction to second progression) was 13.1 months vs. 9.2 months for the maintenance arm vs. the chemotherapy complete-free interval, respectively [25].

Reinduction of the same regimen in the COIN trial controlled the disease for a median of 6 months. The median time to progression was 3 months, and the median number of cycles of reinduction was 2 [26]. The failure-free survival was 8.4 months in the continuous treatment arm vs. 7.4 months in the complete cessation of chemotherapy arm.

In standard chemotherapy, changing the regimen is the logical step at disease progression. For patients treated with a second-line irinotecan-based chemotherapy, the PFS was between 2.5 months and 4.7 months, while oxaliplatin after first-line irinotecan-based chemotherapy was associated with a PFS of 4.2–4.7 months [32].

In a retrospective analysis of 617 patients with mCRC, Loree et al. [33] showed that patients treated with intermittent or maintenance therapy lived longer than did those treated with continuous therapy (40 or 36 vs. 20 months, respectively). They also investigated whether retreatment with the same agent at progression or continuous therapy improved the OS, with evidence in favor of the first group of patients [32]. Logistic regression analysis did not show significance for the type of chemotherapy (irinotecan- or oxaliplatin-based regimens) as an independent prognostic factor for OS.

## Conclusions

Our findings suggest that starting with an irinotecan-based regimen in combination with bevacizumab increases the PFS1 and TTF. Bevacizumab treatment after progression (especially double-dose treatment) is better partnered with the same regimen as that used in the induction phase to improve OS. No definitive conclusions could be drawn for the second-line chemotherapy, with bevacizumab best associated with PFS2.

## Supporting information

S1 File (XLSX)

S1 Data (XLSX)
